# A patient with recurrent pelvic bone metastasis after gastric cancer surgery achieved long-term survival: a case report

**DOI:** 10.3389/fonc.2025.1571265

**Published:** 2025-07-24

**Authors:** Yan Wang, Xiao-Yan Hu, Chao-Qun Wang

**Affiliations:** ^1^ Department of Medical Oncology, Affiliated Dongyang Hospital of Wenzhou Medical University, Dongyang, Zhejiang, China; ^2^ Department of Pathology, Affiliated Dongyang Hospital of Wenzhou Medical University, Dongyang, Zhejiang, China

**Keywords:** gastric cancer, metastasis, pelvic, survival, case report

## Abstract

**Introduction:**

After gastric cancer surgery, solitary bone metastasis is very rare, and repeated pelvic metastasis has not been reported. This article reports a case of recurrent pelvic metastasis after early gastric cancer surgery, in which the patient achieved long-term survival through multiple aggressive surgeries, radiation therapy, and systemic treatment.

**Case Presentation:**

This article reports on a 67-year-old male who presented with “6 days of black stools and 2 days of fatigue.” Gastric endoscopy biopsy indicated gastric cancer, and after completing preoperative examinations, he underwent surgical treatment. The patient experienced recurrent pelvic metastases after gastric cancer surgery and underwent multiple surgeries, combined with radiotherapy and chemotherapy. In the end, he achieved long-term survival of 56 months.

**Conclusions:**

For gastric cancer patients with isolated bone metastases, active surgery in combination with other treatment options can be taken. With the goal of reducing symptoms, improvement in quality of life and survival can be achieved.

## 1 Introduction

In 2022, there were 968,000 new cases of gastric cancer globally, with 660,000 deaths, accounting for 4.9% and 6.8% of all new cases and deaths from malignant tumors (1). Over the past few decades, due to advances in prevention and treatment in most countries, the incidence and mortality rates of gastric cancer have decreased (2–4). However, the mortality rate of gastric cancer remains high, with a poor prognosis for advanced gastric cancer (5).

Bone is not a common site of metastasis for gastric cancer, but the prognosis for gastric cancer bone metastasis is poor (6). Because the bone lesions caused by gastric cancer are mostly osteolytic lesions, and multiple bone lesions are more common, they can lead to the occurrence of skeletal-related events (SREs), resulting in a decrease in the patient’s quality of life and increased treatment difficulties (7). Therefore, it is necessary to detect bone metastasis early. However, bone Emission Computed Tomography (ECT) examination is not included in domestic and international follow-up guidelines, and there is no clear diagnostic and treatment standard for bone metastasis, leading to delays in the detection of bone metastasis and the arbitrariness of treatment.

Given the complexity of treatment for gastric cancer bone metastasis, further research is needed to uncover the potential mechanisms of these metastatic events. By advancing knowledge in this field, our goal is to optimize treatment strategies and improve the prognosis of individuals affected by similar metastases.

## 2 Case description

**Figure 1:**
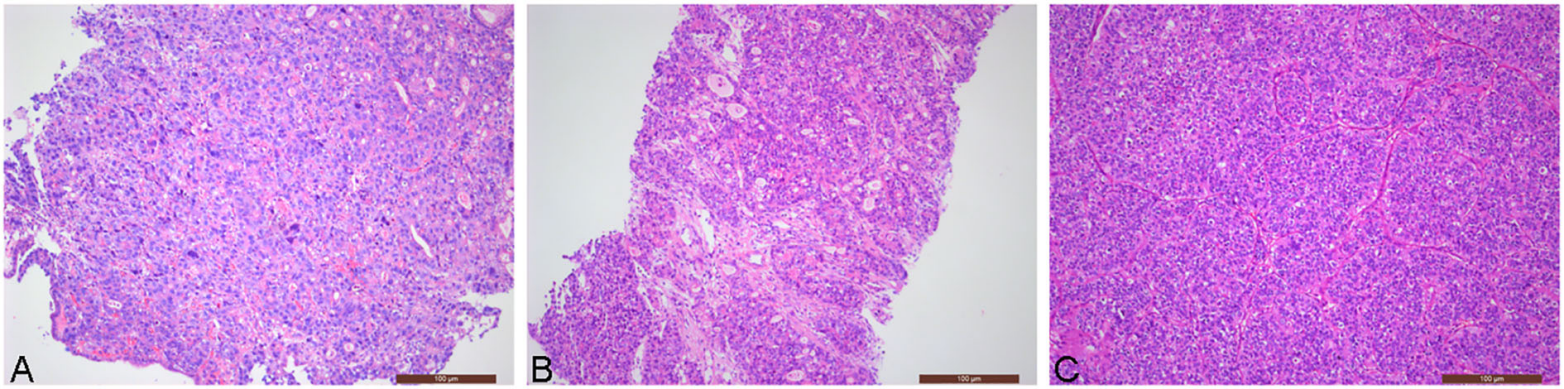
Patient’s pathology images (Hematoxylin and eosin stain, ×100): **(A)** Pathological examination of gastroscopy biopsy revealed poorly differentiated adenocarcinoma; **(B)** Pathological image of left iliac bone mass puncture in November 2020 (gastric adenocarcinoma metastasis); **(C)** Pathological image of left acetabular tumor resection in May 2022 (gastric adenocarcinoma metastasis).

Male patient, 67 years old. Visited on September 23, 2019 for “recurring black stools for 6 days, fatigue for 2 days”. Ultrasound gastroscopy showed a low echogenic mass in the gastric body. Pathological examination of gastroscopy biopsy indicated a malignant tumor, consistent with poorly differentiated adenocarcinoma based on histological morphology (**Figure 1A**). Tumor markers (CEA, CA199, AFP) were all within normal range. Preoperative examinations were completed, and the patient underwent radical gastrectomy for gastric cancer on October 11, 2019. Postoperative pathology results showed: “Limited ulcerative type tubular adenocarcinoma (moderately to poorly differentiated) at the junction of the gastric antrum and body, size 20×18mm; LAUREN classification: intestinal type; infiltration depth: superficial muscle layer; negative margins; lymph node metastasis: 0/17; immunohistochemistry results: HER2 (2+), MSH2 (+), MSH6 (+), PMS2 (+), MLH1 (+)”, staged as T2N0M0 IB stage. The patient did not undergo chemotherapy after surgery.

**Figure 2:**
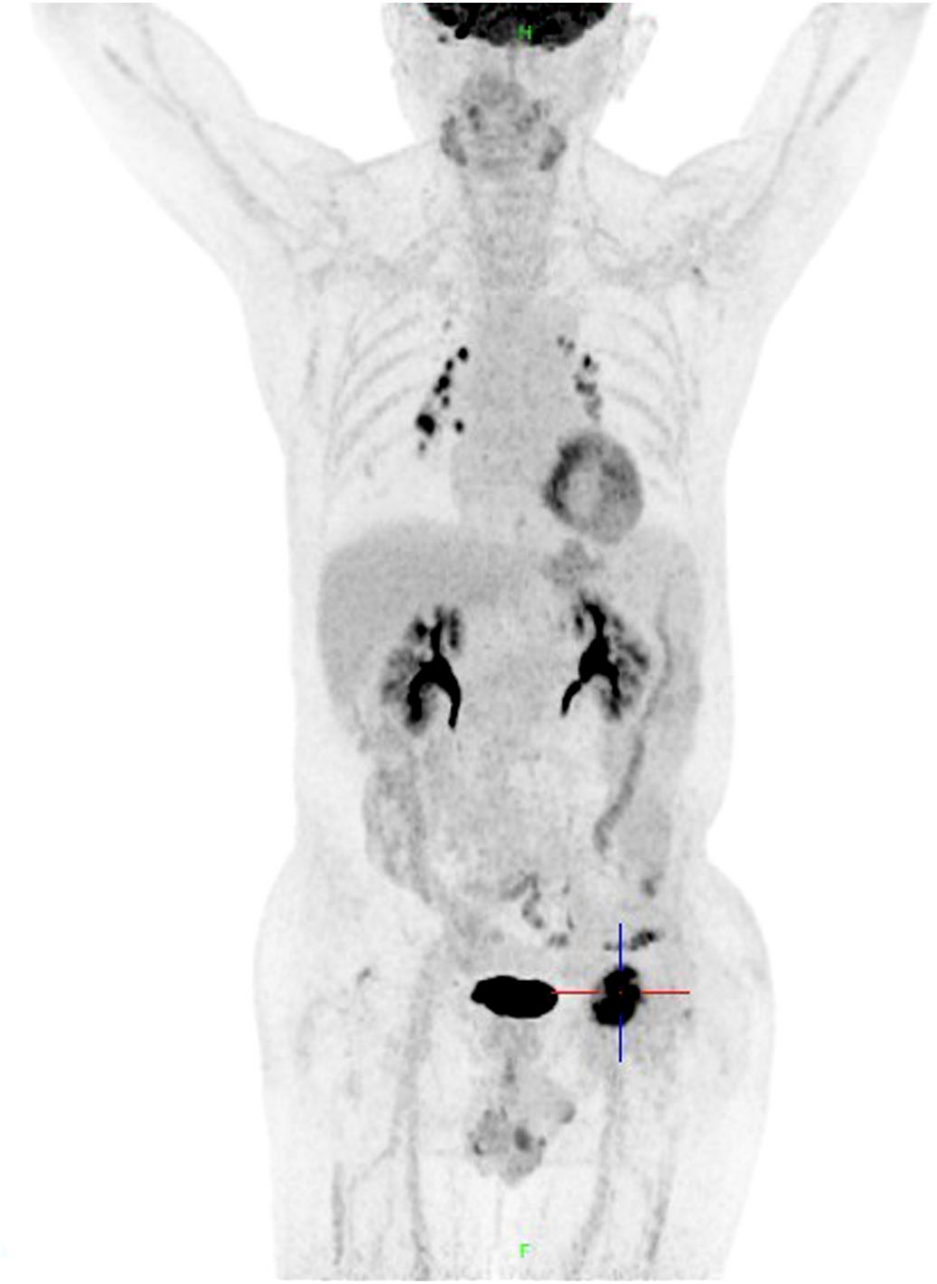
ECT examination in October 2020 showed left iliac bone metastatic tumor.

On October 15, 2020, the patient experienced left hip pain. Chest and abdominal CT scan showed left iliac wing bone destruction with surrounding soft tissue swelling, suggestive of metastatic malignant tumor. ECT examination indicated metastatic bone tumor in the left iliac bone (**Figure 2**). On November 9, 2020, a left iliac bone mass puncture was performed, and the pathology results of the biopsy showed adenocarcinoma, consistent with gastric adenocarcinoma metastasis based on the medical history and immunohistochemistry (**Figure 1B**). Immunohistochemistry results showed CDX2(+), CK20(slightly+), HER2(2+), P53(+), Ki-67(about 40%+). Blood CEA, CA199 were within the normal range, blood calcium was not elevated, LDH was normal, and alkaline phosphatase (ALP) was slightly elevated (128U/L). From November 20 to December 7, 2020, palliative radiotherapy was started for the left hip. On December 12, 2020, a “left hemipelvis tumor resection and reconstruction” was performed, completely removing the tumor tissue. The postoperative pathology suggested “left pelvic tumor, metastatic adenocarcinoma, in line with the medical history, indicating a tendency towards gastric cancer metastasis. The tumor size was 8×5cm, with negative bone margins and surrounding edges. Her-2 gene testing results showed amplification; Mismatch Repair (MMR) testing did not detect Mismatch Repair-deficient (dMMR); Microsatellite Instability (MSI) testing did not detect Microsatellite Instability-High (MSI-H).” On February 22, 2021, treatment with trastuzumab, oxaliplatin, and capecitabine chemotherapy combined with targeted therapy was started. The last treatment was on May 6, 2022, and during the above-mentioned treatment period, regular CT scans did not show tumor recurrence.

On May 20, 2022, the patient visited the orthopedics department due to progressive worsening of left hip pain with limited mobility. A CT scan of the hip indicated bone destruction in the left acetabulum, suggesting possible tumor metastasis. On May 21, 2022, the patient underwent “left acetabular tumor resection and bone reconstruction surgery (palliative surgery)”. Pathological examination results: metastatic or infiltrating adenocarcinoma of the left acetabulum (**Figure 1C**). Immunohistochemistry: CAM5.2 (+), Vimentin (-), CDX2 (+), CK20 (-), CEA (+), HER2 (2+), P53 (+), Ki-67 (40%+). Combining the patient’s medical history and immunohistochemistry results suggest that the source of the cancer is gastric. Postoperative pain improved, and mobility restrictions were alleviated. Starting on June 14, 2022, the patient received chemotherapy with Tegafur, Gimeracil, and Oteracil Potassium Capsules, targeted therapy with Trastuzumab, and immunotherapy with Sintilimab until August 21, 2022. Chemotherapy combined with targeted therapy and immunotherapy is the recommended treatment regimen for HER2-positive advanced gastric cancer according to the Chinese Society of Clinical Oncology (CSCO) Guidelines. Clinical trial data from ORIENT-16 (NCT03745170) confirmed that Sintilimab plus chemotherapy demonstrated benefit in advanced gastric cancer. Considering drug accessibility and financial factors, the patient ultimately chose Sintilimab.

On September 11, 2022, due to worsening pain, an enhanced CT scan revealed multiple bone destruction in the left acetabulum with the formation of soft tissue masses. Considering tumor progression, the chemotherapy regimen was altered. Starting on September 16, 2022, the patient received chemotherapy with albumin-bound paclitaxel, targeted therapy with Trastuzumab, immunotherapy with Sintilimab, and palliative radiotherapy for the left acetabular bone metastasis and soft tissue masses until March 2023, resulting in improved pain.

In early April 2023, the patient experienced worsening pain in the left limb again, and the examination indicated tumor progression in the posterior part of the left acetabulum. As a result, the patient underwent “pelvic lesion excision surgery and total hip arthroplasty revision surgery (palliative surgery)”. Starting on June 12, 2023, the patient received treatment with Vedicetumab, with the last dose administered on August 4, 2023, but discontinued due to significant skin itching. The patient later experienced a decrease in physical strength and appetite, so pain relief treatment was provided, and pain control was stable. A follow-up CT scan on April 6, 2024, showed no tumor recurrence. However, on May 28, 2024, a lung CT scan revealed multiple rib metastases. The patient and family decided to discontinue treatment, and the patient passed away on May 31, 2024.

## 3 Discussion

We reported for the first time a patient with early gastric cancer who had recurrent pelvic metastasis post-surgery, but achieved long-term survival through multiple aggressive surgeries, radiotherapy, and systemic treatment. Gastric cancer is ranked fifth in terms of incidence and mortality globally, with the East Asian region having the highest incidence and mortality rates of gastric cancer in the world (1). In China, the incidence of stomach cancer ranks 5th among malignant tumors, and the mortality rate ranks 3rd (2). Thanks to the progress of endoscopy and imaging examinations in gastric cancer screening and treatment methods, the 5-year overall survival (OS) of gastric cancer has improved (8). The 5-year OS rate for stomach cancer in China increased from 27.4% in 2003–2005 to 35.1% in 2012-2015 (9). However, the prognosis of patients with advanced gastric cancer remains poor, with a median survival of less than 1 year (5). Gastric cancer commonly metastasizes to the liver, peritoneum, lymph nodes, etc., but bone metastasis is rare, with an incidence rate of only 0.9%-2.1% (10). Postoperative isolated bone metastasis in gastric cancer is even rarer, so there are few studies on it. Park et al. reported that out of 1683 postoperative gastric cancer patients, only 5 cases (0.3%) had solitary bone metastasis, with 4 cases being spinal bone metastasis and only 1 case being pelvic bone metastasis (11). However, they did not provide specific treatment and survival information for these 5 patients (11). The most common sites for bone metastasis are the spine, pelvis, and ribs (7, 11). Most gastric cancer patients with bone metastasis experience pain and seek medical attention for it. The patient we reported had postoperative pelvic bone metastasis discovered during a reexamination for pain, and subsequent worsening pain each time indicated tumor recurrence or progression. Patients with bone metastasis after gastric cancer surgery have a very poor prognosis, with a median survival of 6 months (11). The survival period after bone metastasis is related to whether the patient received chemotherapy, ALP levels, tumor differentiation, and whether it is an isolated tumor (7, 12). Therefore, understanding the clinical and pathological characteristics of gastric cancer patients with solitary bone metastasis can provide some clues for clinicians. Studies have shown that gastric cancer patients with larger tumors, lower differentiation, and lymph node metastasis are more likely to develop bone metastasis (11). Additionally, diffuse-type gastric cancer is more likely to develop gastric cancer bone metastasis than intestinal-type gastric cancer (13). Poorly differentiated cancer is the most common pathological type of gastric cancer to develop bone metastasis, and signet ring cell carcinoma, due to its high malignancy, has a much higher rate of bone metastasis than adenocarcinoma (12, 14).

Most patients with bone metastases seek medical attention after experiencing symptoms such as pain, restricted movement, numbness, etc. By the time imaging studies are performed, multiple bone metastases are usually already evident. At this point, patients may also exhibit abnormalities in the hematopoietic system, such as decreased blood cell counts, as well as complications like hypercalcemia and an increased risk of blood clots due to bed rest, making the condition more complex. Typically, patients require a combination of radiation therapy and chemotherapy, but the decline in overall health and abnormalities in the hematopoietic system increase the risks of treatment. Timely detection of occult bone metastases, especially isolated ones, and taking proactive treatment measures can lead to smoother treatment for patients and improve their survival outcomes (15). Unfortunately, ECT examination is not included in routine follow-up examinations. Clinicians often consider bone metastases when patients complain of pain, difficulty in limb movement, or even paralysis, which inevitably delays the discovery of bone metastases and increases the difficulty of treatment. Therefore, early detection of bone metastases is crucial. Although elevated tumor markers, LDH, ALP, and abnormal blood calcium levels have been reported to indicate bone metastases, some patients may not exhibit abnormalities in these markers (16). Like the patient described in this study, his CEA and CA199 levels were normal when bone metastases first appeared, blood calcium level was not elevated, LDH was normal, and ALP was only slightly elevated. Therefore, more sensitive and specific markers or techniques are needed to detect patients who may have bone metastases.

The mechanism of bone metastasis in gastric cancer has not been clarified yet, and some scholars believe it is a process of interaction between tumor cells and the bone microenvironment. Studies have shown that primary tumors may remotely induce the formation of a permissive environment in distant organs to promote and prepare for future metastasis (17). The organotropism of tumor cells towards the bone may be related to the fact that bone is an immune-privileged site. Research has found that bone has a low abundance of cytotoxic T cells and natural killer (NK) cells, and a high proportion of regulatory T cells, which is conducive to the settlement of tumor cells and evasion of immune surveillance (18).

Currently, the treatment for tumor bone metastasis includes systemic anti-tumor therapy and treatment focused on relieving symptoms to prevent bone complications. Although retrospective studies suggest that the prognosis of gastric cancer with bone metastasis is poor (6), prospective studies are lacking due to the rarity of cases, especially with isolated bone metastasis being rare. There is limited stratified analysis of gastric cancer patients with bone metastasis, therefore, no prospective data on the relationship between gastric cancer bone metastasis and prognosis are available. Hence, current guidelines both domestically and internationally do not differentiate in systemic anti-tumor therapy for gastric cancer based on the presence of bone metastasis (19). Treatment focused on relieving symptoms includes surgery, radiation therapy, zoledronic acid or denosumab, and pain management. Data on gastric cancer patients undergoing surgery for bone metastasis is limited, with only a few case reports showing benefits in cases of isolated bone metastasis (15). In this case, the patient underwent three bone metastasis tumor resection surgeries and achieved long-term survival. Particularly, after the third surgery, the patient did not undergo systemic anti-tumor therapy but still had nearly a year of recurrence-free survival, indicating the benefits of surgery for patients with isolated bone metastasis. However, the selection of surgical patients still requires support from larger sample data.

It should be noted that the patient in this article was pathologically staged as IB after gastric cancer surgery. According to the CSCO guidelines, postoperative follow-up is only required to avoid overtreatment (20). However, the European Society for Medical Oncology (ESMO) guidelines recommend adjuvant chemotherapy for IB stage patients (21). We propose that stage IB patients should be stratified based on key risk factors such as tumor type (e.g., signet ring cell carcinoma), histological differentiation (poorly differentiated tumors), and lymph node involvement. Patients exhibiting these high-risk features may benefit from adjuvant chemotherapy despite their IB classification. This individualized approach warrants further exploration to optimize postoperative management and avoid missing potential survival benefits in higher-risk subgroups, while preventing overtreatment in truly low-risk patients.

## 4 Conclusion

We reported a case of a patient with isolated bone metastasis from gastric cancer who underwent 3 surgeries, 2 radiotherapies, combined systemic chemotherapy, targeted therapy, and immunotherapy, and achieved long-term survival of 56 months. Particularly after the third surgery, the patient did not receive systemic anti-tumor treatment and still had nearly 1 year of recurrence-free survival. In conclusion, this case highlights the rare occurrence of recurrent pelvic metastasis after gastric cancer surgery and the possibility of achieving long-term survival through aggressive treatment strategies such as multiple surgeries, radiation therapy, and systemic treatment. It underscores the importance of considering active surgical intervention in combination with other treatment modalities for gastric cancer patients with isolated bone metastases, as it can lead to symptom reduction, improved quality of life, and prolonged survival. Further studies and clinical trials may be warranted to explore the efficacy and potential benefits of such multidisciplinary approaches in managing advanced gastric cancer with bone metastases.

## Data Availability

The original contributions presented in the study are included in the article/supplementary material. Further inquiries can be directed to the corresponding author.
